# Impact of Swift Versus Spray Coagulation on Procedural Outcomes in Gastric Endoscopic Submucosal Dissection

**DOI:** 10.5152/tjg.2026.26081

**Published:** 2026-06-18

**Authors:** Berk Bas, Ömer Küçükdemirci, Altay Kandemir, İsmail Taşkıran, Mehmet Hadi Yaşa, Halil Şahin, Müge Erdem

**Affiliations:** 1 Department of Gastroenterology, Aydın Adnan Menderes University Faculty of Medicine, Aydın, Türkiye; 2 Department of Gastroenterology, Ondokuz Mayıs University Faculty of Medicine, Samsun, Türkiye; 3 Department of Gastroenterology, Liv Hospital, İstanbul, Türkiye; 4 Department of Gastroenterology, İstanbul Aydın University Faculty of Medicine, İstanbul, Türkiye

**Keywords:** Complications, delayed bleeding, electrosurgery, endoscopic submucosal dissection, gastric cancer

## Abstract

**Background/Aims:**

Endoscopic submucosal dissection (ESD) is an established curative treatment for early gastric neoplasms; however, procedure-related bleeding remains a frequent and clinically significant complication. Electrosurgical settings, particularly the choice of coagulation mode, represent potentially modifiable procedural factors that may influence bleeding control and procedure efficiency during gastric ESD. The current study aimed to compare procedure time and procedure-related complications, particularly bleeding, between spray coagulation and swift coagulation modes in patients undergoing gastric ESD.

**Materials and Methods:**

This single-center retrospective cohort study included 194 patients who underwent gastric ESD between 2021 and 2025. Patients were divided into 2 groups according to the electrosurgical coagulation mode used during the procedure: spray coagulation (n = 113) and swift coagulation (n = 81). Procedure time, intraprocedural and delayed bleeding, use of hemostatic forceps, perforation, post-ESD electrocoagulation syndrome (PECS), recurrence, and stricture formation were analyzed and compared between groups.

**Results:**

Procedure time was significantly shorter in the spray coagulation group compared with the swift coagulation group (38.2 ± 20.7 vs. 44.4 ± 19.1 minutes; *P* = .0003). Overall intraprocedural bleeding occurred less frequently in the spray coagulation group (44.2% vs. 75.3%; *P* < .001), and the need for hemostatic forceps was significantly reduced. Severe bleeding events were rare and comparable between groups. Rates of perforation, delayed bleeding, PECS, recurrence, and stricture formation did not differ significantly between groups. Procedural success rates were high and comparable between the groups (93.8% in both groups; *P* = .99). Similarly, en bloc resection rates (92.9% vs. 92.6%; *P* = .94) and complete (R0) resection rates (91.2% vs. 91.4%; *P* = .97) were comparable between the spray and swift coagulation groups.

**Conclusion:**

Spray coagulation during gastric ESD is associated with improved bleeding control and shorter procedure time compared with swift coagulation, without increasing procedure-related complications. Optimization of electrosurgical coagulation mode may enhance procedural efficiency and safety during gastric ESD.

**Main Points**

- Spray coagulation during gastric endoscopic submucosal dissection (ESD) is associated with significantly shorter procedure time compared with swift coagulation.
- Intraprocedural bleeding occurs less frequently with spray coagulation, resulting in a reduced need for hemostatic forceps.
- Rates of severe bleeding, delayed bleeding, perforation, and other procedure-related complications are comparable between spray and swift coagulation modes.
- Optimization of electrosurgical coagulation mode represents a modifiable factor that may improve procedural efficiency and safety during gastric ESD.

## Introduction

Endoscopic submucosal dissection (ESD) has become an established organ-preserving, minimally invasive treatment treatment for early-stage neoplastic lesions of the upper gastrointestinal tract and an effective alternative to surgical resection. In particular, for early gastric cancer, high-grade dysplasia, and selected superficial gastric neoplasms, ESD achieves high en bloc and R0 resection rates while preserving gastric anatomy and function, with favorable long-term oncologic outcomes and improved quality of life compared with surgery.^1^^-^^4^

Recent advances in endoscopic imaging and resection techniques have further improved the diagnosis and management of gastric neoplastic lesions, enabling more accurate lesion characterization and appropriate selection of endoscopic treatment strategies for early gastric lesions.^5^^-^^7^

Bleeding remains one of the most common complications of gastric ESD and is influenced by multiple factors, including lesion size, anatomical location, depth of invasion, patient-related comorbidities, and the use of antithrombotic medications. Beyond these nonmodifiable factors, several procedural variables remain potentially modifiable, such as electrosurgical generator settings, hemostatic strategy, and coagulation mode selection. Optimization of these technical factors may help reduce bleeding-related adverse events and improve procedural efficiency.^8^^-^^10^

ESD relies heavily on electrosurgical energy delivery for both submucosal dissection and hemostasis. Spray and swift coagulation are 2 commonly used electrosurgical modes that differ in voltage profile, tissue contact characteristics, and thermal spread. Spray coagulation is a high-voltage, noncontact mode that enables rapid superficial coagulation of submucosal vessels, whereas swift coagulation delivers continuous low-voltage energy, allowing simultaneous cutting and coagulation with more focal tissue interaction.^11^^-^^13^

These technical differences may have important clinical implications. Excessive thermal injury has been associated with complications such as post-ESD electrocoagulation syndrome (PECS) and perforation, whereas insufficient coagulation may lead to uncontrolled intraprocedural or delayed bleeding, particularly in the highly vascular gastric submucosa.^14^^-^^16^ However, comparative data on the impact of coagulation mode on gastric ESD outcomes remain limited, and no clear consensus exists regarding the optimal electrosurgical strategy.

Most available studies evaluating electrosurgical techniques in ESD are limited by small sample sizes, heterogeneous lesion characteristics, and variability in procedural techniques. As a result, coagulation mode selection in daily practice is often guided by operator preference rather than evidence-based recommendations, highlighting the need for comparative clinical data evaluating its impact on procedure time and bleeding-related outcomes.^17^^,^^18^

The current study aimed to compare procedure time and procedure-related complications, particularly bleeding, between spray coagulation and swift coagulation modes in patients undergoing gastric ESD. In addition, the impact of coagulation mode selection on procedural safety and effectiveness was evaluated, including delayed bleeding, perforation, PECS, recurrence, and stricture formation.

## Materials and Methods

### Study Design and Patient Selection

**Figure 1.**
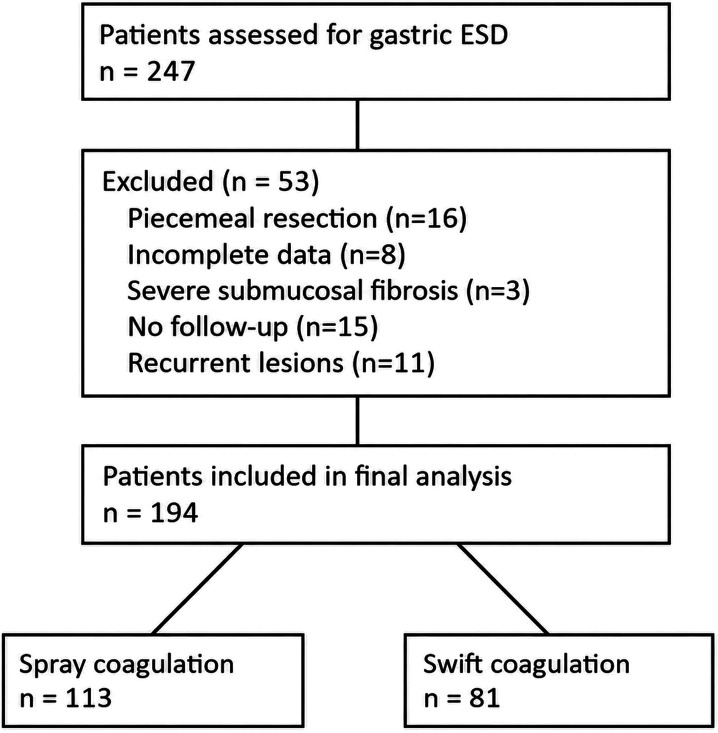
Flowchart of patient selection for gastric endoscopic submucosal dissection (ESD). A total of 247 patients were initially assessed, of whom 53 were excluded based on predefined criteria, including piecemeal resection, incomplete data, severe fibrosis, lack of follow-up, and recurrent lesions. Ultimately, 194 patients were included in the final analysis and categorized according to the electrosurgical coagulation mode used during ESD (spray vs. swift coagulation).

This single-center retrospective cohort study included 194 patients who underwent gastric ESD between January 2021 and December 2024 at a Adnan Menderes University. The study protocol was approved by the local ethics committee Adnan Menderes University (approval date: December 7, 2025; approval number: 334/2025), and the study was conducted in accordance with the principles of the Declaration of Helsinki. All patients were followed according to institutional protocol after the procedure. The mean follow-up duration was 12 months. Scheduled control endoscopy was performed at 3 and 12 months in all patients to assess mucosal healing, detect residual or recurrent lesions, and evaluate procedure-related complications. Subsequent follow-up visits were arranged based on clinical findings and surveillance guidelines (Figure 1).

All procedures were performed at a tertiary referral training center performing more than 300 ESD procedures annually. A senior gastroenterologist with experience of more than 1000 independent ESD procedures both performed and supervised the interventions. In addition, 2 experienced gastroenterologists, each with experience of more than 50 independent gastric ESD procedures, performed procedures during the study period. Furthermore, 4 visiting endoscopists, each having performed more than 50 ESD procedures prior to participation, conducted procedures under the direct supervision of the senior endoscopist. All procedures were either performed or directly supervised by the senior endoscopist to ensure procedural standardization and patient safety. Written informed consent was obtained from all patients before the procedure for the use of their clinical data for research purposes.

Inclusion criteria were as follows:

1. Gastric location of the lesion;
2. ESD performed with curative intent;
3. Clear documentation of the electrosurgical coagulation mode used during the procedure;
4. Age ≥18 years;
5. Regular postprocedural follow-up;
6. First-time ESD performed for the target lesion.

Exclusion criteria were as follows:

1. Piecemeal resection;
2. Incomplete clinical or procedural data;
3. Incomplete lesion resection or aborted procedures;
4. Lack of regular follow-up after ESD;
5. Age <18 years; and
6. ESD performed for recurrent lesions.

### Endoscopic Submucosal Dissection Procedure

Lesion margins were delineated before the procedure using white-light endoscopy and, when necessary, narrow-band imaging. The selection of coagulation mode was based on operator preference. During the early phase of the study period, the senior endoscopist observed improved hemostatic control with spray coagulation while performing peroral endoscopic myotomy. Following these observations, spray coagulation was gradually adopted during gastric ESD as an alternative electrosurgical strategy for submucosal vessel coagulation. Consequently, both spray and swift coagulation modes were used in routine clinical practice during the study period.

Importantly, the choice of coagulation mode was not determined by predefined patient or lesion characteristics. Instead, the mode was selected by the operator at the beginning of the procedure based on procedural preference and maintained throughout the dissection to ensure technical consistency and minimize intraprocedural variability.

Submucosal injection was performed using a Voluven-based solution with indigo carmine to enhance visualization of the submucosal layer and facilitate safe dissection. The same solution was used in all patients to maintain procedural consistency.

### Coagulation Modes and Definitions

Spray coagulation is a high-voltage, noncontact electrosurgical mode that produces superficial thermal coagulation and is commonly used for rapid coagulation of submucosal vessels. In the current study, spray coagulation was primarily used for vessel coagulation and control of intraprocedural bleeding during gastric ESD.

Swift coagulation is a continuous electrosurgical mode that provides combined cutting and coagulation effects. This mode was primarily used during submucosal dissection to allow simultaneous tissue dissection and hemostasis.

### Electrosurgical Generator and Settings

All procedures were performed using a high-frequency electrosurgical generator (ERBE VIO® 3, ERBE Elektromedizin GmbH, Tübingen, Germany). Spray coagulation was applied using Effect 2-3 at 40-60 W, whereas swift coagulation was applied using Effect 3-4 at 30-50 W. Minor adjustments within these ranges were permitted according to intraprocedural findings and operator judgment.

### Endoscopic Equipment and Accessories

All procedures were performed using high-definition upper gastrointestinal endoscopes (Fujinon series, FUJIFILM Corporation, Tokyo, Japan) equipped with a water-jet function and a large working channel. A transparent distal attachment cap was used in all procedures to improve visualization and facilitate submucosal dissection.

Mucosal incision and submucosal dissection were performed using dedicated ESD knives (Micro-Tech Endoscopy, Nanjing, China). When bleeding could not be adequately controlled with the ESD knife, monopolar hemostatic forceps (Boston Scientific Corporation, Marlborough, MA, USA) were used for hemostasis. Injection needles were used for submucosal injection, and hemoclips were applied when necessary.

### Hemostatic Forceps Use

The use of hemostatic forceps was predefined and systematically recorded. Hemostatic forceps were used for the management of active intraprocedural bleeding when bleeding could not be adequately controlled with the ESD knife. Only hemostatic forceps applications performed for active bleeding control were counted in the hemostatic forceps use variable. Prophylactic coagulation of visible submucosal vessels to prevent bleeding was not included in the hemostatic forceps count. The total number of bleeding-related hemostatic forceps applications per procedure was recorded and analyzed.

### Procedure Time

Procedure time was defined as the interval from the start of mucosal incision following lesion marking to the completion of en bloc resection of the lesion.

### Outcome Measures

Primary outcomes were procedure time and procedure-related bleeding.

The severity of intraprocedural bleeding was classified according to the Endoscopic Resection Bleeding (ERB) classification system. ERB-0 was defined as the absence of active bleeding during dissection, typically after prophylactic coagulation of visible vessels. ERB-c1 (minor bleeding) was defined as bleeding that was easily controlled endoscopically without affecting the patient’s vital signs and without requiring blood transfusion. ERB-c2 (moderate bleeding) was defined as bleeding that prolonged the procedure and required additional endoscopic hemostatic interventions but remained controllable endoscopically. ERB-c3 (major bleeding) was defined as severe bleeding that could be controlled endoscopically but required blood transfusion or resulted in prolonged hospitalization. Prophylactic coagulation of visible submucosal vessels performed to prevent bleeding was not considered intraprocedural bleeding.

Secondary outcomes included delayed bleeding within 14 days after ESD, perforation, PECS, recurrence, and stricture formation.

### Procedural success

Procedural success was defined as successful completion of ESD with en bloc removal of the target lesion without conversion to surgery or additional endoscopic resection techniques. En bloc resection referred to removal of the lesion in a single piece, whereas complete (R0) resection was defined as en bloc resection with histologically tumor-free lateral and vertical margins confirmed by pathological examination. Intraprocedural bleeding was defined as bleeding requiring endoscopic hemostatic intervention. Delayed bleeding was defined as clinically significant hemorrhage occurring more than 24 hours after ESD that required endoscopic intervention, blood transfusion, or hospitalization. Perforation was defined as a transmural defect detected endoscopically or radiologically. PECS was defined as localized abdominal pain, fever, or leukocytosis without radiologic evidence of perforation.

### Statistical Analysis

Continuous variables were expressed as mean ± SD or median (minimum–maximum), and categorical variables as number and percentage. Continuous variables were compared using the Student’s *t*-test or Mann–Whitney *U*-test, and categorical variables using the chi-square test or Fisher’s exact test.

Multivariate logistic regression analysis was performed to identify independent factors associated with bleeding. Statistical analyses were conducted using Statistical Package for the Social Sciences (SPSS) SPSS Statistical Package for the Social Sciences (SPSS) version 26.0 (IBM SPSS Corp.; Armonk, NY, USA) , and a *P* value <.05 was considered statistically significant.

## Results

### Baseline Demographic and Clinical Characteristics

During the study period, 247 patients were evaluated for gastric ESD. Fifty-three patients were excluded because of piecemeal resection (n = 16), incomplete procedural data (n = 8), severe submucosal fibrosis (n = 3), lack of regular follow-up (n = 15), and ESD performed for recurrent lesions (n = 11). Ultimately, 194 patients were included in the final analysis.

Of these, 113 patients were treated using spray coagulation and 81 using swift coagulation. Baseline demographic and clinical characteristics of the study population are summarized in Table 1. The mean age was similar between the spray and swift coagulation groups (61.3 ± 12.9 vs. 60.1 ± 9.9 years, respectively; *P* = .334). The proportion of male patients was also comparable between groups (51.3% vs. 53.1%; *P* = .338).

The distribution of ASA scores did not differ significantly between the groups (*P* = .056). Similarly, the use of anticoagulant or antiplatelet medications was comparable between the spray and swift coagulation groups (16.8% vs. 13.6%, respectively; *P* = .680).

Lesion-related characteristics are presented in Table 2. The mean lesion size was slightly larger in the spray coagulation group compared with the swift coagulation group (3.32 ± 1.10 cm vs. 3.05 ± 0.97 cm; *P* = .019).

Pathological diagnoses were distributed similarly between groups (*P* = 0114). Tubular adenoma was the most common diagnosis in the spray coagulation group, whereas hyperplastic polyps were more frequent in the swift coagulation group.

Lesion location did not differ significantly between groups (*P* = .128). In both groups, the majority of lesions were located in the antrum, followed by the corpus and cardia.

### Procedural Characteristics

Procedural outcomes are summarized in Table 3. The mean procedure time was significantly shorter in the spray coagulation group compared with the swift coagulation group (38.2 ± 20.7 vs. 44.4 ± 19.1 minutes; *P* = .0003).

The use of hemostatic forceps differed markedly between the 2 groups. The mean number of hemostatic forceps applications was significantly lower in the spray coagulation group compared with the swift coagulation group (1.49 ± 1.87 vs. 3.04 ± 2.52; *P* < .001). Similarly, the proportion of patients requiring at least 1 coagrasper application was significantly lower in the spray coagulation group (46.0% vs. 75.3%; *P* < .001).

Submucosal injection volume was also lower in the spray coagulation group compared with the swift coagulation group (19.8 mL vs. 25.5 mL; *P* = .012).

Procedural success rates were high and comparable between the groups. Procedural success was achieved in 93.8% of patients in both groups (*P* = .99). Likewise, en bloc resection rates (92.9% vs. 92.6%; *P* = .94) and complete (R0) resection rates (91.2% vs. 91.4%; *P* = .97) were similar between the spray and swift coagulation groups (Table 3).

The distribution of bleeding severity according to the ERB classification is shown in Table 3. The absence of intraprocedural bleeding was more frequent in the spray coagulation group compared with the swift coagulation group (54.9% vs. 24.7%; *P* < .001).

Among patients who experienced bleeding, the distribution of bleeding severity (minor, moderate, or severe) did not differ significantly between groups (*P* = .297). Severe bleeding events were rare in both groups.

Other procedure-related complications are presented in Table 3. Perforation rates were low and comparable between groups (3.5% in the spray coagulation group vs. 2.5% in the swift coagulation group, *P* = .997).

PECS was observed exclusively in the swift coagulation group (7.4%) and was not recorded in the spray coagulation group (*P* = .012). Rates of delayed bleeding, recurrence, and stricture formation were low and did not differ significantly between the 2 groups (Table 3).

The distribution of coagulation mode according to operator, pathological diagnosis, and procedural year is presented in Table 4. Coagulation mode selection did not significantly differ among operators (*P* = .805), indicating that the choice of electrosurgical mode was not associated with a specific endoscopist. Similarly, no significant difference was observed in coagulation mode distribution according to pathological diagnosis (*P* = .114). However, a significant temporal shift in coagulation mode preference was observed across the study period (*P* < .001). Spray coagulation was rarely used during the early years of the study but became progressively more frequent in later years, ultimately replacing swift coagulation as the preferred technique in the final study year.

Multivariable logistic regression analysis was performed to identify independent predictors of intraprocedural bleeding (Table 5). Among the evaluated variables, procedure time was identified as the only independent factor significantly associated with bleeding. Specifically, longer procedure time was associated with an increased risk of bleeding (OR 1.11, 95% CI 1.06-1.15, *P* < .001). Age, sex, lesion size, ASA score, anticoagulant or antiplatelet use, and lesion location (cardia, corpus, bulbus, or fundus compared with antrum), were not significantly associated with bleeding risk.

To further investigate factors associated with bleeding severity, an ordinal logistic regression analysis was performed among patients who developed intraprocedural bleeding (Table 5). In this analysis, higher ASA score was independently associated with increased bleeding severity (OR 4.73, 95% CI 1.38-16.17, *P* = .013). Age, sex, lesion size, procedure time, anticoagulant use, and lesion location were not significantly associated with bleeding severity.

**Figure 2.**
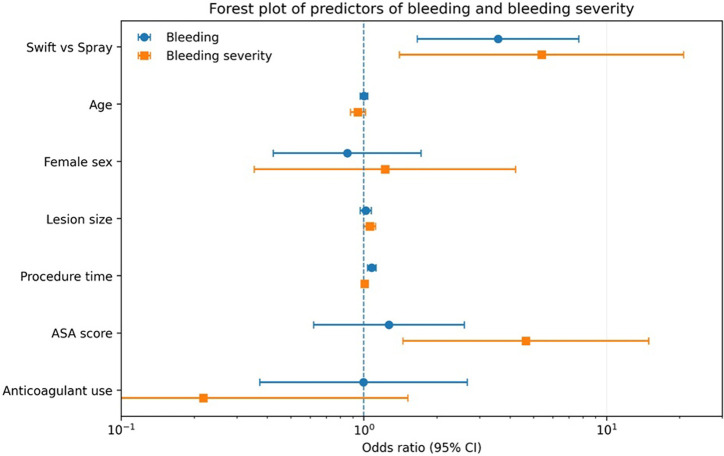
Forest plot demonstrating the association between clinical and procedural variables and the risk of intraprocedural bleeding and bleeding severity during gastric ESD. Odds ratios (ORs) with 95% CIs were calculated using logistic regression analysis. The vertical dashed line represents the null value (OR = 1). Variables included age, sex, lesion size, procedure time, ASA score, anticoagulant use, and coagulation mode (swift vs. spray).

The results of both multivariable analyses are presented in the forest plot (Figure 2), which summarizes the odds ratios and CIs for predictors of intraprocedural bleeding and bleeding severity.

Overall, these results indicate that spray coagulation was associated with a lower incidence of intraprocedural bleeding and a reduced need for hemostatic interventions.

## Discussion

In this single-center retrospective study, the use of spray coagulation during gastric ESD was associated with significantly shorter procedure time, lower rates of intraprocedural bleeding, and a reduced need for hemostatic forceps compared with swift coagulation. Importantly, these advantages were achieved without an increase in major procedure-related complications, including perforation, delayed bleeding, PECS, recurrence, or stricture formation. Procedural success and en bloc resection rates were high and comparable between the 2 groups.

Bleeding remains one of the most frequent and clinically relevant adverse events during gastric ESD, with direct implications for procedure time, technical difficulty, and patient outcomes. Intraprocedural bleeding has been reported in approximately 20%-60% of gastric ESD cases, whereas delayed bleeding occurs in approximately 2%-10% of cases in previous studies. In the current cohort, the overall bleeding rate was within the upper range of published series; however, this relatively higher incidence is likely explained by the inclusion of minor oozing-type bleeding events in the definition of intraprocedural bleeding. Even mild bleeding may impair visualization, interrupt submucosal dissection, and necessitate additional hemostatic maneuvers, thereby prolonging the procedure.^19^^-^^21^

The favorable hemostatic profile of spray coagulation may be explained by its noncontact energy delivery and more homogeneous superficial thermal effect. This mode appears to allow effective coagulation of submucosal vessels while minimizing tissue adherence and uncontrolled bleeding. Such an effect may be particularly beneficial in the stomach, where the richly vascularized submucosal layer can rapidly obscure the dissection plane when bleeding occurs.^22^^-^^24^ In contrast, swift coagulation provides more focal energy delivery at the tissue interface, which may increase the likelihood of vessel injury or insufficient hemostasis when larger or tortuous submucosal vessels are encountered. In support of this interpretation, Ishikawa et al reported that spray coagulation reduced the need for hemostatic forceps during gastric ESD, reinforcing the concept that electrosurgical mode selection is a clinically relevant and modifiable procedural factor.^25^^26^

Consistent with this, the need for hemostatic forceps was significantly lower in the spray coagulation group, indicating more effective intraprocedural bleeding control. This finding is clinically relevant not only because it reflects improved hemostasis, but also because fewer bleeding-related interruptions may contribute directly to improved procedural efficiency. Indeed, procedure time was significantly shorter in the spray coagulation group, despite the fact that lesion size was not smaller in this group. This observation suggests that improved bleeding control may translate into shorter and technically smoother procedures through better visualization of the submucosal layer, fewer device exchanges, and reduced interruption of dissection.^27^^-^^29^

Notably, procedural success, defined by en bloc and R0 resection rates, was high and comparable between groups, with an overall success rate of 94%. Large gastric ESD series from experienced centers have reported en bloc resection rates of approximately 90%-95%. Consistent with these reports, the findings suggest that coagulation mode selection does not appear to influence resection quality but rather affects intraprocedural bleeding control and procedural efficiency.^30^^-^^32^ Similarly, the higher frequency of multiple knife use in the swift coagulation group may reflect increased carbonization or device wear during repeated attempts at hemostasis, representing an additional procedural and economic disadvantage.

The overall complication profile was favorable in both groups. Rates of perforation, delayed bleeding, recurrence, and stricture formation were low and comparable, consistent with previously published gastric ESD series reporting perforation rates of approximately 1%-5% and delayed bleeding rates of 2%-10% in experienced centers. These findings indicate that the improved hemostatic performance of spray coagulation was not achieved at the expense of reduced safety.^33^^,34,15^ Although the broader thermal dispersion of spray coagulation might theoretically raise concern for deeper mural injury, the results do not support an increased risk of thermal injury–related adverse events with this mode.

PECS deserves particular attention. Previous gastric ESD studies have reported PECS rates ranging from approximately 2% to 10%, depending on lesion characteristics and procedural factors. In the current study, PECS was observed exclusively in the swift coagulation group (7.4%), whereas no cases were detected in the spray coagulation group. Although spray coagulation might theoretically be expected to increase the risk of transmural thermal injury because of its broader thermal effect, the findings did not support this assumption. One possible explanation is that the shorter procedure time and improved bleeding control observed with spray coagulation may have reduced cumulative thermal exposure and tissue injury. Increasing institutional experience and improved procedural efficiency over time may also have contributed to the relatively low overall incidence of PECS in the current cohort.^35-37^

Because the selection of coagulation mode was not randomized, the potential influence of temporal and operator-related factors should also be considered. The analysis demonstrated that spray coagulation was increasingly preferred in the later years of the study period. However, this temporal shift could not be directly attributed to operator experience alone, as procedures were performed by endoscopists with heterogeneous levels of experience throughout the study period, including a senior endoscopist with extensive ESD experience and several operators with lower but still substantial procedural exposure. Furthermore, similar bleeding-related findings were observed among endoscopists with different levels of experience, suggesting that the benefit of spray coagulation is unlikely to be explained solely by a chronological learning-curve effect.

Multivariable analysis provided additional insight into the determinants of bleeding. Procedure time was the only independent factor associated with the occurrence of intraprocedural bleeding, indicating that longer and presumably more technically demanding procedures are at higher risk of bleeding. In contrast, age, sex, lesion size, ASA score, antithrombotic use, and lesion location were not independently associated with bleeding occurrence. Among patients who developed bleeding, ASA score was the only independent predictor of bleeding severity, suggesting that when bleeding does occur, its clinical severity may be influenced more by the patient’s overall systemic condition than by lesion-related or procedural factors. These findings highlight the importance of careful procedural planning and vigilant hemostatic management, particularly in prolonged procedures and in patients with greater comorbidity burden.

The current study had several notable strengths. It reflects real-world clinical practice in a high-volume tertiary referral center with substantial advanced endoscopy experience. It directly compares 2 commonly used electrosurgical strategies and focuses on a modifiable procedural factor with immediate clinical relevance. In addition, detailed procedural data, including bleeding severity and the need for hemostatic interventions, were systematically recorded, allowing a comprehensive evaluation of bleeding-related outcomes. All procedures were performed under expert supervision within a structured training environment, further supporting the reliability of the findings.

The current study had several limitations. It was a single-center retrospective study, which may limit the generalizability of the findings to institutions with different patient populations, operator experience, and procedural practices. Although the sample size is comparable to that of many gastric ESD series, it may still be insufficient to detect small but clinically meaningful differences in infrequent adverse events. Increasing institutional experience during the study period may have contributed to improved procedural efficiency and bleeding control; however, the observation of similar findings across endoscopists with different experience levels suggests that the benefit of spray coagulation cannot be attributed solely to the learning curve. In addition, long-term follow-up was not uniformly available, limiting comprehensive assessment of late local recurrence and metachronous lesions. Detailed histopathological variables that may influence procedural difficulty, such as submucosal fibrosis, microvascular density, and depth of invasion, were not systematically recorded and therefore could not be included in subgroup analyses. Finally, because of the retrospective design, operator-dependent technical variables—including dissection strategy, knife selection, and traction techniques—could not be fully evaluated. Despite these limitations, the findings suggest that spray coagulation represents a favorable and safe electrosurgical strategy for gastric ESD, improving intraprocedural bleeding control without increasing major complications and thereby facilitating smoother submucosal dissection and overall procedural performance.

In conclusion, in this single-center retrospective study, spray coagulation during gastric ESD was associated with reduced intraprocedural bleeding, decreased need for hemostatic forceps, and shorter procedure time compared with swift coagulation. These benefits were achieved without compromising resection quality or increasing major adverse events. Although the influence of increasing institutional experience cannot be completely excluded, the overall findings suggest that coagulation mode selection is a clinically meaningful and modifiable procedural factor in gastric ESD. Further prospective, multicenter studies are needed to confirm these findings and refine evidence-based recommendations for electrosurgical strategy selection.

## Figures and Tables

**Table 1.** Baseline Demographic and Clinical Characteristics of Patients

Variable	Spray Coagulation (n = 113)	Swift Coagulation (n = 81)	*P*
Age, years	61.3 ± 12.9 (63)	60.1 ± 9.9 (62)	.334
Male sex, n (%)	58 (51.3)	43 (53.1)	.338
ASA score distribution, n (%)	0.056
	
ASA I	64 (56.6)	54 (66.7)	.158
ASA II	36 (31.9)	25 (30.9)	.882
ASA III	13 (11.5)	2 (2.5)	.018
Anticoagulant/antiplatelet use, n (%)	19 (16.8)	11 (13.6)	.680

Data are presented as mean ± standard deviation (median) for continuous variables and as number (percentage) for categorical variables. Continuous variables were compared using the Student’s *t*-test or Mann–Whitney *U*-test, depending on data distribution. Categorical variables were compared using the chi-square test or Fisher’s exact test, as appropriate.

ASA, American Society of Anesthesiologists.

**Table 2.** Lesion Characteristics

Variable	Spray Coagulation (n = 113)	Swift Coagulation (n = 81)	*P*
Lesion size, cm	3.32 ± 1.10 (3.2)	3.05 ± 0.97 (2.8)	.019
Pathological diagnosis, n (%)			.114
Hyperplastic polyp	27 (23.9)	31 (38.3)	
NET	19 (16.8)	14 (17.3)	
Tubular adenoma	32 (28.3)	15 (18.5)	
GIST	10 (8.8)	2 (2.5)	
Adenocarcinoma	17 (15.0)	9 (11.1)	
Tubulovillous adenoma	2 (1.8)	3 (3.7)	
Other	6 (5.3)	7 (8.6)	
Lesion location, n (%)			.128
Cardia	18 (15.9)	13 (16.0)	
Corpus	25 (22.1)	11 (13.6)	
Antrum	63 (55.8)	50 (61.7)	
Fundus	7 (6.2)	7 (8.6)	

GIST, gastrointestinal stromal tumor; NET, neuroendocrine tumor.

**Table 3.** Procedural Outcomes and Procedure-related Complications

Variable	Spray Coagulation (n = 113)	Swift Coagulation (n = 81)	*P*
Procedural characteristics			
Procedure time, minutes	38.2 ± 20.7 (33)	44.4 ± 19.1 (40)	.0003
Coagrasper use count	1.49 ± 1.87 (0)	3.04 ± 2.52 (3)	<.001
Coagrasper use ≥1, n (%)	52 (46.0)	61 (75.3)	<.001
Submucosal injection volume, mL	19.8	25.5	.012
Procedural success, n (%)	106 (93.8)	76 (93.8)	.99
En bloc resection, n (%)	105 (92.9)	75 (92.6)	.94
Complete (R0) resection, n (%)	103 (91.2)	74 (91.4)	.97
Intraprocedural bleeding severity			
No bleeding	62 (54.9)	20 (24.7)	<.001
Minor bleeding (c1)	44 (38.9)	44 (54.3)	
Moderate bleeding (c2)	6 (5.3)	12 (14.8)	
Severe bleeding (c3)	1 (0.9)	3 (3.7)	
Procedure-related complications			
Perforation	4 (3.5)	2 (2.5)	.997
Post-ESD electrocoagulation syndrome	0 (0)	6 (7.4)	.012
Delayed bleeding	1 (0.9)	2 (2.5)	.565
Recurrence	1 (0.9)	1 (1.2)	1
Stricture	0 (0)	0 (0)	–

ESD, endoscopic submucosal dissection.

**Table 4.** Distribution of Coagulation Mode According to Operator, Pathology, and Procedural Year

Variable	Category	Spray n (%)	Swift n (%)	Total (n)	*P*
Operator	Operator 1	57 (56.4)	44 (43.6)	101	
	Operator 2	31 (58.5)	22 (41.5)	53	
	Operator 3	25 (62.5)	15 (37.5)	40	.805
Pathology	Hyperplastic polyp	27 (46.6)	31 (53.4)	58	
	NET	19 (57.6)	14 (42.4)	33	
	Tubular adenoma	32 (68.1)	15 (31.9)	47	
	GIST	10 (83.3)	2 (16.7)	12	
	Adenocarcinoma	17 (65.4)	9 (34.6)	26	
	Tubulovillous adenoma	2 (40.0)	3 (60.0)	5	.114
Procedure year	2020	0 (0.0)	17 (100.0)	17	
	2021	1 (3.2)	30 (96.8)	31	
	2022	35 (57.4)	26 (42.6)	61	
	2023	28 (77.8)	8 (22.2)	36	
	2024	49 (100.0)	0 (0.0)	49	<.001
Total					

**Table 5.** Multivariable Analysis of Predictors of Intraprocedural Bleeding and Bleeding Severity

Variable	Bleeding OR	95% CI	*P*	Bleeding severity OR	95% CI	*P*
Age	1.00	0.97-1.04	.91	0.93	0.87-1.00	.064
Female sex	0.96	0.49-1.89	.90	1.96	0.60-6.33	.26
Lesion size	0.99	0.94-1.04	.57	1.03	0.97-1.09	.34
Procedure time	1.11	1.06-1.15	<.001	1.02	0.99-1.05	.25
ASA score	1.16	0.58-2.33	.67	4.73	1.38-16.17	.013
Anticoagulant use	1.00	0.37-2.69	.99	0.28	0.04-1.93	.20

Multivariable logistic regression analysis was performed to identify independent predictors of intraprocedural bleeding. In addition, ordinal logistic regression analysis was conducted among patients who developed bleeding to evaluate predictors of bleeding severity. Results are presented as odds ratios (OR) with 95% CI.

ASA, American Society of Anesthesiologists.

## Data Availability

The data that support the findings of this study are available on request from the corresponding author.
